# Paroxysmal Cold Hemoglobinuria in an Adult with Respiratory Syncytial Virus

**DOI:** 10.1155/2018/7586719

**Published:** 2018-11-13

**Authors:** Ryan Leibrandt, Kenneth Angelino, Monique Vizel-Schwartz, Ilan Shapira

**Affiliations:** ^1^Department of Medicine, Mount Sinai Beth Israel, New York, NY, USA; ^2^Division of Hematology and Medical Oncology, Department of Medicine, Mount Sinai Hospital, New York, NY, USA; ^3^Division of Hematology and Medical Oncology, Department of Medicine, Mount Sinai Beth Israel, Mount Sinai Hospital, New York, NY, USA

## Abstract

Paroxysmal cold hemoglobinuria (PCH) is a rare form of cold autoimmune hemolytic anemia first discovered in the early 20th century in adults with tertiary syphilis. Today, it is more commonly seen in children as a life-threatening anemia during a viral upper respiratory tract infection (URI). Although respiratory syncytial virus (RSV) has previously been reported to cause PCH in a child, herein we present the first documented case in an adult. The Donath–Landsteiner (DL) test, the diagnostic test for PCH, was positive on two separate occasions. The patient was treated successfully with warming and avoidance of cold temperatures. To facilitate identification of this rare entity by clinicians, we include a discussion about the pathophysiology, diagnosis, and treatment of PCH.

## 1. Case Presentation

A 64-year-old female presented to the emergency department (ED) in February due to “wine-colored urine.” This was preceded by two weeks of viral upper respiratory illness (URI) symptoms, including fevers, chills, cough, headache, and malaise. Two days prior to presentation, she received azithromycin with no improvement in her symptoms. She had no past medical history and took no medications prior to this illness. Her family history was notable for a daughter with systemic lupus erythematosus.

Laboratory data included hemoglobin 11.7 (11.7–15.0 g/dL), total bilirubin 3.4 (0–1.2 mg/dL), and direct bilirubin 0.8 (<0.9 mg/dL). Urine was red and hazy with a moderate amount of blood. The sediment contained 4 + bacteria, 0–3 red blood cells (RBCs), and 11–25 white blood cells (WBCs) per high-power field (HPF). It was negative for nitrites, leukocyte esterase, urobilinogen, and bilirubin. Chest X-ray and CT abdomen/pelvis enhanced with oral and intravenous contrast did not reveal any acute pathology. She was started on nitrofurantoin for suspected urinary tract infection and discharged home. Urine cultures later showed no evidence of bacterial growth.

Four days later, she returned to the ED due to low-grade fevers, weakness, and persistence of urine discoloration. Physical exam was notable for scleral icterus and jaundice. Her hemoglobin decreased to 8.9 g/dL with total bilirubin 2.9 mg/dL, direct bilirubin 0.7 mg/dL, AST 84 (<36 U/L), ALT 42 (<46 U/L), alkaline phosphatase 75 (38–126 U/L), reticulocytes 4.4 (0.7–2.8%), lactate dehydrogenase (LDH) 2,397 (100–220 U/L), and undetectable haptoglobin. The peripheral smear showed polychromasia, occasional nucleated red blood cells but no schistocytes or red blood cell agglutination. Urine was amber-colored containing a large amount of blood, moderate amount of bilirubin and urobilinogen <2.0 mg/dL. Urine staining for hemosiderin was negative twice. Coagulation studies were normal. The direct antiglobulin test (DAT) was positive for C3d and negative for IgG. Testing for cryoglobulins and cold agglutinins was negative. Mycoplasma serology was positive for IgG and negative for IgM. Testing for HIV, hepatitis A, B, and C, Epstein–Barr virus, and antinuclear antibody was all negative. Glucose-6-phosphate dehydrogenase assay was adequate. RSV nucleic acid was detected via real-time polymerase chain reaction testing from a nasopharyngeal swab (Xpert® Xpress Flu/RSV). Testing for the Donath–Landsteiner (DL) antibody was positive, confirming the diagnosis of paroxysmal cold hemoglobinuria.

Management consisted of warming the patient and avoiding exposure to cold temperatures. She did not require any transfusions and by hospital day two, her hemoglobin stabilized and urine discoloration resolved over the ensuing two weeks. Two months after initial presentation, the repeat DL test was positive, along with DAT positive for C3d and negative for IgG. The follow-up hemolysis panel was notable only for mild elevation of LDH (245 U/L) with normal haptoglobin and reticulocyte count. Six months after initial presentation, the DL test was negative and hemoglobin was normal.

## 2. Discussion

Paroxysmal cold hemoglobinuria (PCH) is a rare form of cold autoimmune hemolytic anemia characterized by a biphasic, polyclonal IgG. When PCH was first discovered, it was described as a chronic relapsing condition in adults with tertiary syphilis [1]. Today, the acute form of PCH in children is most commonly described with a median age of five years (range, 1–82) [2] comprising 30–40% of all pediatric cases of autoimmune hemolytic anemia [3]. Certain viruses have been implicated in acute episodes of PCH in children, including measles, mumps, varicella, cytomegalovirus, EBV, influenza, parvovirus B19, coxsackievirus, and adenovirus. Only one other case report by Santos Malavé et al. documents RSV causing PCH in a child [4]. To our knowledge, the case presented above is the first report of PCH triggered by RSV in an adult.

While bacterial etiologies in addition to syphilis have been reported (*Mycoplasma*, *H. influenzae*, *Klebsiella*, and *E. coli*), an inciting infectious etiology is rarely discovered [5]. The mechanism of how a virus or bacterium induces PCH is poorly understood. One proposed theory is that viruses alter glycoproteins in the RBC membrane which then stimulate autoantibody formation. Another theory posits that molecular mimicry between self-antigens and foreign antigens results in formation of cross-reactive antibodies [6]. Noninfectious etiologies of PCH such as non-Hodgkin's lymphoma (NHL) and chronic lymphocytic leukemia (CLL) have also been implicated. In a patient with NHL, hemolysis resolved only after the patient received chemotherapy. Investigators were able to demonstrate that the DL antibodies originated from lymphoma cells [7, 8]. In a patient with CLL, the DL antibody showed specificity for the i-antigen on the RBC surface, as opposed to the more common p-antigen [9]. The poor understanding of the etiology of PCH further complicates a clinician's ability to make the diagnosis.

The classic clinical scenario of PCH is the passage of red-brown urine preceded by viral URI symptoms. The antibody typically develops within one week of a URI and can persist for months despite resolution of hemolysis [10]. Accompanying physical exam findings can include jaundice, scleral icterus, pallor, abdominal pain, and fever [11]. Lab findings are consistent with hemolysis: indirect hyperbilirubinemia, decreased haptoglobin, low complement, increased LDH, and reticulocytosis. There will be evidence of hemoglobinuria as was evident in our case, with urinalysis showing blood but few to no RBCs. Hemosiderinuria is also characteristic but typically develops 3-4 days after the onset of hemolysis which could explain why this was negative in our patient [12]. Although infrequently seen, the pathognomonic finding on the peripheral smear is erythrophagocytosis by neutrophils.

The diagnosis of PCH is confirmed by the presence of the DL antibody, a biphasic, polyclonal IgG that binds to the surface of RBCs, most commonly the p-antigen, in the colder temperature of the extremities. The antibody fixes the first two components of the complement cascade and then dissociates upon rewarming, resulting in complement-mediated intravascular hemolysis. Due to this biphasic nature, DAT is negative for IgG and positive for anti-C3. In the differential diagnosis of PCH, cold agglutinin disease has the same DAT results but a negative DL test. Furthermore, IgM will be positive, and the peripheral smear will show RBC agglutination.

Julius Donath and Karl Landsteiner discovered this unique mechanism of hemolysis in 1904, after which the gold standard diagnostic test today was named [1]. This test can be performed by either directly using whole blood or indirectly using separated serum. In the direct test, two blood specimens are collected and immediately incubated at 37°C. The test specimen is cooled to 0°C for one hour and then rewarmed to 37°C for thirty minutes while the control specimen is maintained at 37°C for the entire procedure. The supernatant serum of the chilled sample should demonstrate signs of hemolysis, indicating a positive test [13]. A potential cause of a false-negative result is low levels of serum complement due to their consumption during a prior hemolytic event. In addition, RBCs can be coated with C3dg which protects from complement-mediated lysis. For these reasons, the indirect test has a higher sensitivity compared to the direct test.

In the indirect test, the patient's serum is combined with normal serum for an additional source of complement, while maintaining a temperature of 37°C. ABO-compatible p-positive RBCs are then added, and the test specimen is incubated to 0°C. Visible hemolysis in the test specimen and the absence of hemolysis in the control indicates a positive DL test. The indirect test still has the potential for false negatives due to the transient nature of the DL antibody after resolution of the initial hemolytic episode.

Variations of the DL test exist to increase its sensitivity. These include pretreatment of RBCs with 1% papain, an enzyme that exposes more p-antigen sites on the RBC membrane leading to more antibody binding. Due to its technical difficulty and variable interpretation, the DL test is often performed incorrectly leading to false-negative results and an underreporting of PCH [14]. In a review of the literature from 1996 to 2016, Zeller et al. found only nine reported cases.

Given the self-resolving nature of acute PCH, management primarily involves avoidance of cold temperatures. Warming the patient, in addition to warming all ingestions or intravenous fluids, can prevent continued hemolysis [15]. Depending on the severity of the anemia, transfusions can be given, ideally with warmed blood lacking the p-antigen. For chronic PCH, cold avoidance remains the mainstay of treatment. Glucocorticoids have historically been used for refractory anemia but there is insufficient evidence to demonstrate any benefit [16]. Other therapies such as cyclophosphamide, rituximab, and eculizumab have been employed; however, data to support their efficacy are mixed. While C5 is routinely targeted in the treatment of paroxysmal nocturnal hemoglobinuria, upstream inhibition of C3 is a theoretical means to prevent both intravascular and extravascular hemolysis. Targeting this part of the complement cascade could potentially be applicable to PCH as well; however, clinical trials are needed to evaluate the safety and efficacy of this treatment strategy [17].

The mainstay of treatment for PCH is supportive care and close observation. As our patient's DL test remained positive two months after presentation, she was monitored for continued signs of hemolysis until the DL test returned negative four months later. Although her hemoglobin remained stable, unrecognized PCH can result in life-threatening anemia. Therefore, clinicians should be aware of this diagnosis as its prompt recognition can lead to simple, life-saving interventions.
